# Objectively-Measured Sedentary Time and Self-Reported Prescription Medication Use Among Adults: A Pilot Study

**DOI:** 10.3390/pharmacy12060186

**Published:** 2024-12-10

**Authors:** Ciarra A. Boyne, Tammie M. Johnson, Lindsay P. Toth, Michael R. Richardson, James R. Churilla

**Affiliations:** 1 Department of Clinical and Applied Movement Sciences, Brooks College of Health, University of North Florida, Jacksonville, FL 32224, USA; l.toth@unf.edu (L.P.T.); m.richardson.136601@unf.edu (M.R.R.); j.churilla@unf.edu (J.R.C.); 2 Institute of Public Health, College of Pharmacy and Pharmaceutical Sciences, Institute of Public Health, Florida Agricultural and Mechanical University, Tallahassee, FL 32307, USA; tammie.johnson@famu.edu

**Keywords:** prescription medication, sedentary time, sedentary behavior, accelerometers, drugs

## Abstract

While previous research has linked physical activity (PA) with lower prescription medication consumption, limited evidence has investigated sedentary time (ST) as a major contributor to medication use, despite ST’s known association with chronic disease and mortality risk, even when PA volume is considered. This study aimed to examine the independent associations between objectively measured ST, patterns of sedentary bouts, and self-reported prescription medication use among adults ≥25 years of age. Thirty-two participants reported the number and type of medications they were currently prescribed and wore an accelerometer continuously on their hip for seven days to detect their ST. Poisson regression analysis was used to assess how average daily ST, sedentary bout frequency, and sedentary bout duration influenced medication use. The results revealed a significant association between greater ST and higher medication consumption. Specifically, each additional hour of ST per day, each sedentary bout, and each one-minute increase in bout duration were linked to a 66% (PR 1.66; 95% CI 1.25–2.19; *p* < 0.001), 36% (PR 1.36; 95% CI 1.12–1.64; *p* < 0.01), and 9% (PR 1.09; 95% CI 1.03–1.16; *p* < 0.01) higher prevalence of prescription medications, respectively. These findings suggest that higher ST is associated with a greater prevalence of using prescription medications in adults.

## 1. Introduction

Noncommunicable diseases or chronic diseases have become a leading public health concern both in the United States (U.S.) and worldwide. These diseases rank among the top causes of mortality around the world. According to the World Health Organization (WHO) [1], approximately three-fourths of all global deaths can be attributed to chronic diseases. For example, cardiovascular disease (CVD), cancer, and diabetes alone contribute to 17.9 million, 9.3 million, and 2.0 million global annual deaths, respectively. In the U.S., the burden is similarly high, with nearly 52% of adults diagnosed with at least one chronic condition as of 2018 [2]. In 2020, CVD and cancer resulted in 19 and 10 million deaths, respectively, while nearly 13% of Americans were diagnosed with diabetes between 2017 and 2020, with rates continuously climbing [3].

As the prevalence of chronic diseases steadily increases, there is growing concern about the role of sedentary behavior (SB) and sedentary time (ST) as major factors in these negative health issues. Evidence consistently demonstrates that excessive SB or prolonged ST is linked to an augmented risk of cardiovascular disease, diabetes, cancer, obesity, and both cardiovascular and all-cause mortality [4,5,6,7,8,9,10]. Notably, these relationships typically remain significant despite regular participation in moderate to vigorous physical activity (MVPA). A meta-analysis in 2013 by Chau et al. [11] suggested that participation in MVPA may not fully mitigate the mortality risk associated with greater ST. Investigators revealed a 34% increase in all-cause mortality risk when adults reported sitting for 10 h per day after adjusting for physical activity (PA).

Prescription medication use has also been shown to be independently related to poor health outcomes such as CVD, diabetes, metabolic syndrome, and mortality [12,13]. When examining modifiable factors related to prescription medication use, previous evidence has primarily focused on the inverse relationship between PA volume and prescription medications [14,15]. There is a paucity of evidence regarding ST and prescription medication use, although ST is another important predictor of risk for chronic diseases and is independently related to mortality regardless of PA volume [10,11].

Historically, questionnaires or surveys have been used to obtain estimates of total ST. However, using objective methods to monitor ST, such as accelerometers, otherwise known as wearable activity monitors, helps to minimize self-report bias and allows for examination of sedentary bout patterns as they occur in the free-living environment, which may be significant contributors to certain health outcomes. Additionally, when reviewing various methods of capturing ST (i.e., self-reporting via questionnaires versus activity monitors), Healy et al. [16] determined there are mixed outcomes for survey methods in overestimating or underestimating ST. Therefore, the aims of this study were to (1) examine the association between objectively measured ST via accelerometers and self-reported prescription medication use in adults ≥25 years of age and (2) examine the independent associations of sedentary bout length and frequency with medication consumption in adults.

## 2. Methods

This pilot study was conducted from October 2022 to April 2023 on the University of North Florida (UNF, Jacksonville, FL, USA) campus and the surrounding northern Florida area. The final sample consisted of 32 adults, aged 25–65 years, who met the following criteria: (1) adult men and women (≥25 years of age); (2) not pregnant; (3) no upper or lower body injuries that would limit normal daily movement; and (4) no diagnosis of intellectual disabilities or cognitive impairment. The institutional review board (IRB) of UNF approved all testing and examination procedures in October 2022, and all participants signed an informed consent.

Participants were asked to continuously wear an ActiGraph GT9X activity monitor (ActiGraph LLC, Pensacola, FL, USA) on their waist while performing their normal daily routines. They were instructed to wear the activity monitor continuously for 24 h a day (capturing all behavior during waking and sleep hours) for seven consecutive days and to only remove it during water-based activities (e.g., bathing, swimming, sauna). Participants were included in the final analysis if they wore the activity monitor for at least ten hours a day on at least four out of the seven days. The seven-day protocol period was utilized as it has been found to be 90% reliable in assessing ST, based on agreement with subjective ST assessments, according to Matthews et al. [17]. Although Matthews et al. recommended a seven-day protocol, they did not specify a minimum wear time, and there is no universal standard. Therefore, the minimum wear time of 10 or more hours per day was based on the widely accepted minimum wear time described by Troiano et al. [18] for the measurement of PA. Furthermore, according to Airlie et al. [19], a minimum wear time criterion of at least eight hours a day is warranted to obtain reliable PA and ST estimates in older adults. During this seven-day protocol period, participants were also asked to record their sleep times and times of monitor removal on two provided logs. Since sleep is not considered SB, it has been recommended that sleep logs be implemented to mitigate the risk of misclassifying sleep as SB [20]. All study participants took part in the questionnaire and activity monitor protocol. A control group was not included due to the nature of a small-sample pilot study.

The primary independent variable was ST, which was assessed objectively via the primary accelerometer of the ActiGraph GT9X activity monitor. Once raw accelerometer data were obtained, they were converted to activity counts per minute (cpm) via the ActiLife software (version 6.13.4, ActiGraph LLC., Pensacola, FL, USA), ActiGraph’s actigraphy data analysis software platform. An activity count is a proprietary unit of measure for physical behaviors that quantifies acceleration within a time interval or “epoch”, with one epoch usually being 10–60 s long [21]. For the present study, the ActiGraph GT9X monitors were initialized to collect data at 30 Hz, and the data were reported in 60 s epochs.

Once the data were converted to cpm, the total ST (minutes per week) from ActiLife were converted to hours per week. For practical purposes, this information was then converted to average ST hours per day for each participant, creating the sedentary time variable, which was analyzed as a continuous variable. According to Troiano et al. [18], sedentary activity is defined as any activity with 0–99 cpm. For the sedentary bout frequency, the total number of sedentary bouts per week from ActiLife was converted to the average number of sedentary bouts per day. The average length of time (minutes) in all sedentary bouts across the seven-day period was obtained from ActiLife and utilized for the sedentary bout duration. Both the sedentary bout frequency and duration were analyzed as continuous variables. According to the Freedson 1998 bout parameters, a sedentary bout is defined as an activity with 0–99 cpm for at least 10 consecutive minutes [22].

Biological sex, age, race/ethnicity, education, waist circumference, and PA were controlled for in this study to prevent any potential confounding. Age was divided evenly into four categories: 25–34, 35–44, 45–54, and ≥55 years of age. This was assessed utilizing participants’ dates of birth obtained from the Demographics questionnaire, administered via Qualtrics, which was created for the purpose of this study. Race/ethnicity was divided into three categories: White (including Hispanic White), Black (including Hispanic Black), and Other (including “prefer not to say”). This was self-reported in the Demographics questionnaire. Education was categorized into two levels: some college or less (which included less than high school and some high school, high school graduate, and some college including AA degree) and college graduate. This was also self-reported in the Demographics questionnaire. Waist circumference (WC) was dichotomized (healthy/unhealthy) based on the World Health Organization classifications for obesity and disease risk [23]. An unhealthy WC was defined as ≥102 cm (40 in) and ≥88 cm (35 in) for men and women, respectively. WC was measured using a tape measure just above the uppermost lateral border of the right ilium to the nearest centimeter. Lastly, PA was dichotomized as meeting/exceeding the PA guidelines or not meeting the PA guidelines based on the 2018 U.S. Department of Health and Human Services (DHHS, Washington, DC, USA) PA Guidelines [24]. Meeting the PA guidelines was defined as engaging in ≥150 m/wk of moderate or ≥75 m/wk of vigorous PA. Physical activity was objectively measured via the ActiGraph GT9X activity monitor. Using the 2008 Troiano adult cut-points [18], light activity is defined as 100–2019 cpm, moderate activity is defined as 2020–5998 cpm, and vigorous activity is defined as 5999+ cpm. Therefore, moderate to vigorous physical activity (MVPA) would be any activity ≥2020 cpm. This variable was created using the total minutes of MVPA per week obtained from ActiLife.

The dependent variable in this study was prescription medication use. Prescription medication use was operationalized as the number of prescription medications an individual took in the past 30 days. This information was obtained through a medical and prescription medication history questionnaire via Qualtrics that utilized similar components of the National Health and Nutrition Examination Survey (NHANES, Hyattsville, MD, USA) [25]. Within this questionnaire, participants were asked to identify the number, brand or generic medication names, and reasonings/diagnoses for taking the prescribed medication(s). The Ambulatory Care Drug Database System [26] from the National Center for Health Statistics (NCHS, Hyattsville, MD, USA) was utilized to classify medications by their therapeutic drug class. This is based on the same system that the NHANES uses, which is Lexicon Plus^®^, a proprietary database of Cerner Multum, Inc. (Denver, CO, USA). This system uses the Multum drug classification to categorize drugs by their therapeutic class.

Once the raw accelerometer data were downloaded with ActiLife, the data cleaning process was completed. This consisted of verifying the non-wear time with the times of monitor removal that were recorded on the wear time log and the measured sleep times with the sleep log. Once the sleep times were verified, all accounts of sleep were marked as non-wear time for the final analysis.

The data in this study were analyzed utilizing IBM SPSS version 26.0 [27]. SPSS was used to conduct variable recodes and data coding validation. The final analysis excluded over-the-counter drugs and all types of oral contraceptives, vitamins, and dietary supplements unless they were prescribed to treat a specific noncommunicable diagnosis or condition. This was decided upon since prescription medication use is a predictor of chronic disease prevalence and general health status [28,29,30], which can help indicate which diseases were more common among the final sample.

Poisson regression analysis was used to quantify the relationship between the number of prescription medications an individual was prescribed based on the average hours of ST per day (significance was established at *p* = 0.05). Separate Poisson regression analyses were also used to predict how the number of prescriptions changed based on the average sedentary bout frequency and duration (significance set at *p* = 0.05). Linear regression models were adjusted for biological sex, age, race/ethnicity, education, waist circumference, and PA. Three models were generated using a forward selection process based on the results of the Poisson regression analysis. Model 1 was a crude model; Model 2 adjusted for demographic factors, including biological sex, age, race/ethnicity, and education as well as WC, and Model 3 adjusted for Model 2 covariates and PA. The prevalence ratios were used to examine the associations between ST and prescription medicine use. Post-hoc power analysis was performed utilizing G*Power version 3.1 [31].

## 3. Results

Table 1 summarizes the sample and ST characteristics when stratified by biological sex. The median age of the participants was 31 years (IQR 27.0–50.6), with 68.8% being women. There were no black participants (including Hispanic Black) who volunteered for this study, and more than three-fourths of the participants were white (including Hispanic White). Additionally, more than three-fourths of the participants were college graduates. About 80% of participants were classified as having a healthy WC, which was defined as <102 cm (40 in) and <88 cm (35 in) for men and women, respectively. Two-thirds of the participants met or exceeded the PA guidelines with a median of 219 min (IQR 106.5–333.8) of MVPA per week.

Among the 32 participants, the sample was evenly dispersed between prescription medication use, where 15 participants (47%) reported taking at least one prescription medication in the past 30 days (reported medication range 1–15). The median number of medications was zero (IQR 0.0–2.0). When stratified by biological sex, women had greater medication use in which 50% of women and 40% of men consumed a prescription drug. When analyzing medication types, antidepressants were the most prevalent, with 40% of participants reporting the use of at least one antidepressant medication. Thyroid drugs and central nervous system stimulants were also highly prevalent, with 33% and 26% of participants, respectively, taking at least one of these drugs. Other reported drugs included anti-inflammatory agents, beta-blockers, proton pump inhibitors, skeletal muscle relaxants, calcium channel blockers, anticonvulsants, antiarrhythmics, and anxiolytics. Participants were sedentary for approximately 76 h per week and 11 h per day in which women were seen to be more sedentary than men. Participants also had 134 sedentary bouts per week and 19 bouts per day, where each bout lasted approximately 23 min.

Table 2 summarizes the sample characteristics when stratified by ST (<11.38 h/day and >11.38 h/day). This threshold was established when examining percentiles of the number of prescription medications and average sedentary hours per day. The 60th percentile is where at least one medication and 11.38 h of ST were observed. Additionally, at the 60th percentile, participants were seen to be taking at least one medication when having approximately 20 sedentary bouts per day and bouts lasting at least 23 min.

The sample size (*N* = 32) allowed for the examination of the relationship between ST and prescription medication use, with a power of 0.87 for the crude model and 0.99 for the fully adjusted model (Model 3). Crude analysis revealed that there was a 29% increase in the expected number of prescriptions for each extra hour of sedentary time per day (PR 1.29; 95% CI 1.08–1.55; *p* = 0.01) (Table 3). Following adjustment for biological sex, age, race/ethnicity, education, and waist circumference, ST remained statistically significant, with a 70% increase in the expected number of prescriptions (PR 1.70; 95% CI 1.29–2.26; *p* < 0.001) for each one-hour increase in ST. Due to such a large change in prevalence from the crude model to Model 2, a pairwise examination of the covariates was performed to explore any possibilities of interactions. Using a forward-selection approach, WC caused a shift in the prevalence ratio from 1.29 to 1.55. However, there was no statistically significant interaction between WC and ST (*p* = 0.48). In the fully adjusted model (adjusted for the Model 2 covariates and PA), there was a 66% increase in the expected number of prescription medications for each hour of ST per day (PR 1.66; 95% CI 1.25–2.19; *p* < 0.001).

Adults (35–44 years) and (45–54 years) had a higher expected number of prescription medications compared to the referent group (25–34 years). Adults (35–44 years) exhibited almost a threefold increase in the expected number of prescription medicines (PR 2.61; 95% CI 1.12–6.09; *p* < 0.05), and adults (45–54 years) had a fourfold increase in the expected number of medications (PR 3.86; 95% CI 1.68–8.88; *p* < 0.05). However, the relationship was not seen for older adults (≥55 years). A statistically significant association among different race/ethnicity groups was also observed. Individuals with other races/ethnicities besides white (including Hispanic White), which was the referent group, had an 84% lower expected number of prescriptions (PR 0.16; 95% CI 0.04–0.58; *p* < 0.05). Lastly, participants classified as having unhealthy WC had a fourfold increase in the expected number of prescriptions they were taking (PR 4.28; 95% CI 1.77–10.30; *p* < 0.05) compared to participants with healthy WC.

After conducting separate Poisson regression analyses utilizing the fully adjusted models, which adjusted for biological sex, age, race/ethnicity, education, WC, and PA, there was a 36% (PR 1.36; 95% CI 1.12–1.64; *p* < 0.01) and 9% (PR 1.09; 95% CI 1.03–1.16; *p* < 0.01) increase in the expected number of medications for each extra bout of ST per day (i.e., frequency) and each one-minute increase in the average time (i.e., duration), respectively (Table 4). There was an attempt to add total ST with the sedentary bout frequency and bout duration variables in two separate models; however, this was not statistically significant. Similar to total ST, adults (35–44 years) and (45–54 years) had a greater expected number of medications compared to the referent group (25–34 years). Adults (35–44 years) had a prevalence ratio of 2.62 (95% CI 1.18–5.78; *p* < 0.05) and 3.26 (95% CI 1.44–7.38; *p* < 0.05) for the bout frequency and duration, respectively, compared to the youngest age group (adults 25–34 years). Adults (45–54 years) had a prevalence ratio of 4.76 (95% CI 1.95–11.63; *p* < 0.05) and 2.93 (95% CI 1.26–6.81; *p* < 0.05) for the bout frequency and duration, respectively, compared to the youngest age group (adults 25–34 years). Adults who were of a race/ethnicity other than white (including Hispanic White) exhibited a 78% lower expected number of prescriptions (PR 0.22; 95% CI 0.06–0.76; *p* < 0.05) for every bout of ST per day. Additionally, adults classified as having unhealthy WC had an 8.5-fold increase (PR 8.50; 95% CI 2.62–27.63; *p* < 0.05) in the expected number of prescriptions compared to adults with healthy WC for every bout of ST per day. Adults who also did not meet the PA recommendations exhibited a twofold increase in the expected number of prescription medications (PR 2.41; 95% CI 1.23–4.73; *p* < 0.05) compared to adults who met the PA guidelines for each extra bout of ST per day. Borderline significance (*p* = 0.05) was seen among both race/ethnicity and education regarding the duration of a sedentary bout. Adults who were of other races/ethnicities and were college graduates compared to adults who were white (including Hispanic White) and had some college or less had a 70% (PR 0.30; 95% CI 0.09–1.01) and 59% (PR 0.41; 95% CI 0.17–1.01) lower expected number of medications, respectively, for each one-minute increase in the average time of a sedentary bout.

Another critical finding was the examination of sedentary breaks, which are non-sedentary periods between two sedentary bouts, and determining that there was a 4% (PR 0.96; 95% CI 0.93–0.99; *p* < 0.05) decrease in the expected number of prescription medications for each one-minute increase in the average duration of a sedentary break. This was analyzed by conducting a separate Poisson regression using the fully adjusted model, which adjusted for biological sex, age, race/ethnicity, education, WC, and PA. This was a significant decrease in prevalence compared to sedentary bout duration and prescription medication use (PR 1.09; 95% CI 1.03–1.16; *p* < 0.01). Using the same methodology to examine the effects of sedentary break frequency versus break duration, Poisson regression analysis revealed a 35% (PR 1.35; 95% CI 1.12–1.64; *p* < 0.01) increase in the expected number of prescription medications for each extra sedentary break per day, which only slightly changed from the association of sedentary bout frequency and prescription medication use (PR 1.36; 95% CI 1.12–1.64; *p* < 0.01).

## 4. Discussion

To the best of our knowledge, this is the first study to exclusively examine the association between ST and prescription medications independent of PA utilizing an objective measure of ST. Our findings suggest that extended ST is associated with an increase in the expected number of prescription medications. Moreover, more frequent sedentary bouts throughout the day and a longer average time of sedentary bouts were both significantly associated with a greater expected number of prescription medications in our sample of 32 adults (≥25 years of age). Participants were sedentary for approximately 11 h a day. This aligns with a review of cross-sectional studies done by Bauman et al. [32], where investigators found that 25% of adults reported sitting for ≥11 h per day.

Almost half of the participants were prescribed at least one medication. In a study by Kantor et al. [33] examining trends in prescription medication use, they identified 18 most common medicines used by U.S. adults. The present study had 7 out of the 18 medicines that were most prescribed. This included antidepressants, thyroid drugs, anti-inflammatory agents, and anxiolytics, among others.

Currently, there is limited evidence regarding the relationship between ST variables and prescription medication use among adults. Previous studies have only investigated the associations between PA and prescription medications with only a brief discussion of sedentary individuals. One cross-sectional study by Bertoldi et al. [14] investigated the correlation between PA and prescription medicine utilization among Brazilian adults aged 20 years or older. Investigators found that less activity was correlated to higher medication use, and specifically, sedentary individuals had a 23% augmented risk for medication utilization compared to the most active individuals. For this study, PA and SB were assessed utilizing a shortened version of the International Physical Activity Questionnaire (IPAQ), which has been validated to obtain and compare PA and SB estimates across multiple countries. The main findings of this study were congruent with our results despite different methods of assessing PA and SB (i.e., IPAQ questionnaire versus accelerometer). However, it has been revealed that the IPAQ may lead to underestimation of ST and an increased risk of recall bias compared to objective accelerometer measurements [34]. Additionally, accelerometers can capture free-living patterns of SB, which allows for more accurate estimates and greater diversity of SB. Two studies have examined the association between PA and medicine utilization using objectively measured ST [15,35]. A study in 2012 by Silva et al. [15] revealed a 55% increase in medication usage among sedentary individuals (classified as <6000 steps per day in this study) after adjusting for many variables, including sitting time (average hours per day) when examining the relationship in 271 older Brazilian women. In the present study, there was a 66% increase in the expected number of prescription medications for each extra hour of ST per day. Silva et al. assessed PA through the number of steps taken using a Yamax Digi-walker pedometer worn on the waist. Investigators revealed that 15% of participants were insufficiently active; this was more than doubled in the present study, where 34% were insufficiently active or classified as not meeting the PA recommendations. However, this may be attributed to how PA was assessed (step count versus moderate to vigorous minutes of PA). A subsequent cross-sectional study by Bielemann et al. [35] examined PA and polypharmacy (i.e., concomitant use of five or more medicines) specifically in Brazilian older adults (ages 60 and up). There was a significant inverse association seen between the lowest tertile of PA and the greatest prevalence of prescription medicines. All three studies demonstrate that individuals who are sedentary or insufficiently active may be taking more medications.

In the present study, participants had a higher expected number of medications if they were white (including Hispanic White), older (between the ages of 35–54 years), and had a higher WC. Greater prescription medication use among white persons may be attributed to socioeconomic status, better access to care, type of health insurance coverage, and more drug coverage with health insurance plans [36]. For example, about 25–29% of minorities received assistance for prescription medication costs from government-assisted programs such as Medicaid and were less likely to have private insurance coverage from employers. Furthermore, the higher prevalence of medication use among older adults was consistent with a population-based study by Bardel et al. [37], who examined prescription medication use among women in Sweden. Investigators showed that women who were 45–54 years old had a twofold increase in odds of using more prescriptions compared to women who were 35–44 years old (referent group), whereas in our study, adults (45–54 years old) had almost a fourfold increase in the expected number of medications consumed compared to our youngest age group (25–34 years old). Evidence has shown that prescription medication use increases with age due to multimorbidity and an aging population where life expectancy exceeds 75 years old in many countries [38]. Another study by Kelly et al. [39] also had similar demographic findings that investigated the relationship between a healthy lifestyle and mortality risk after stratification of medication use, specifically no polypharmacy, polypharmacy, and hyperpolypharmacy. Within their study, they revealed that sedentary individuals had higher medication usage, and higher proportions of participants with unhealthy WC were present in the polypharmacy and hyperpolypharmacy groups. In the present study, participants with higher WC had a fourfold increase in the expected number of prescription medications.

The current study also revealed possible implications for sedentary behavior patterns (i.e., sedentary bouts and breaks) regarding frequency and duration. Frequent sedentary bouts and longer bout durations were both associated with greater prescription medication usage. Additionally, longer sedentary breaks may be more effective in attenuating medication burden compared to more frequent breaks. This finding is consistent with a Finnish cross-sectional study by Farrahi et al. [40] that analyzed cardiometabolic factors of adults based on sedentary time patterns and breaks. Their study revealed that the two groups (“Breakers” and “Shortened sitters”) with the most sedentary breaks and least amount of sedentary time had more favorable cardiometabolic outcomes (i.e., lower adiposity, lipids, and insulin sensitivity levels) compared to the group with the highest amount of accumulated sedentary time. Additionally, the Breakers, which participated in longer breaks with more MVPA between sedentary bouts, displayed greater favorable differences in these cardiometabolic markers compared to the Shortened sitters.

Sedentary time may be linked to more prescription medication use because it is highly correlated with many adverse health outcomes such as cardiovascular disease (CVD), diabetes, cancers (breast, colon, colorectal, and endometrial cancer), obesity, metabolic syndrome, and cardiovascular and all-cause mortality [4,5,6,7,8,9,10]. It is also linked to various physiological factors that may increase one’s risk for chronic disease. For instance, ST has been linked to elevated plasma triglycerides, diminished lipoprotein lipase activity, reduced insulin sensitivity, changes in cardiac output and total peripheral resistance, reduced HDL-cholesterol levels, lower bone mineral densities, and higher WC [7]. Many of these factors can increase one’s risk of dyslipidemia, hypertension, diabetes mellitus, hyperglycemia, hyperinsulinemia, insulin resistance, chronic inflammation, osteoporosis, and obesity.

Because ST can be used to predict disease burden and mortality, the U.S. has included brief ST recommendations in the 2018 DHHS PA Guidelines for Americans [24]. However, these guidelines are specific to PA and are insufficient to recommend a specific threshold for ST. It is recommended to reduce SB and break up extended periods of ST with light PA or movement breaks. Other countries, such as the United Kingdom and Australia, also have nonquantitative guidelines like the U.S. guidelines [41,42,43]. Contrastingly, Canada and Korea have more quantified recommendations. In 2020, Canada released the first-ever 24-h Movement Guidelines recommending no more than eight hours of ST a day, three hours of recreational screen time, and breaking up bouts of ST as frequently as possible for all adults [44]. Additionally, the Korean guidelines suggest that individuals have no more than two hours per day of sedentary leisure time [45]. With more evidence emerging regarding the health impact of ST and with the present study demonstrating greater medication use, thus the higher prevalence of chronic diseases among sedentary individuals, we hope this can contribute to advocating for more refined ST guidelines in the U.S.

The present study has several inherent limitations due to the study design. These limitations include using self-reported data for prescription medication usage, which may be subject to recall bias and a possible social desirability effect. Furthermore, there are some adherence concerns with wearing the ActiGraph (e.g., participants forgetting to put it back on after removing it for showering). However, if participants wore it for at least ten hours a day for at least four days that week, they were included in the final analysis. Additionally, our sample size did not allow for a diverse sample. Due to our study’s observational study design, there needs to be larger experimental studies utilizing more diverse samples in terms of age, race/ethnicity, education, and more medication types/chronic conditions. If reproduced on a larger scale, it may be important to consider using validated questionnaires electronically to obtain self-reported estimates of ST and to compare them to the results of the accelerometers. For instance, the Sedentary Behavior Questionnaire (SBQ) has been validated and proven to be reliable in adults, according to Rosenberg et al. [46]. Using electronically accessible activity or sleep logs could also enhance the feasibility of the study and improve participant compliance if the study were to be replicated on a larger scale. One example is the Consensus Sleep Diary (CSD), which has been recommended by Dietch et al. [47] to monitor total sleep time in adults.

## 5. Conclusions

Our findings suggest that prolonged daily ST, more frequent sedentary bouts, and longer durations of sedentary bouts are associated with an increase in the expected number of prescription medications taken. These relationships remain significant after adjustment of biological sex, age, race/ethnicity, education, waist circumference, and MVPA. Future studies should aim to develop specific ST cut-points further and advocate for their inclusion in the U.S. DHHS PA guidelines to align with other international recommendations.

These findings postulate potential significant clinical implications of reducing total ST as well as incorporating longer breaks from extended periods of ST to possibly alleviate the national disease burden and attenuate the reliance on medications. While increasing participation in regular PA is beneficial to improve health outcomes, there are other various strategies that can be implemented to mitigate the adverse effects of excessive sitting. Daily adjustments such as taking standing or walking breaks during work hours, incorporating more movement into daily routines (e.g., using stairs, performing household chores, walking pets, or parking farther from destinations), investing in standing desks or walking pads (especially in large corporations), and minimizing excessive screen time can all help significantly reduce the need for medications by addressing individuals’ sedentary lifestyles.

## Figures and Tables

**Table 1:** Sample and Sedentary Time Characteristics Stratified by Biological Sex.

	All (*N* = 32)	Men (*n* = 10)	Women (*n* = 22)
**Prescribed Medications (%)**	46.9%	40.0%	50.0%
**Age (years)**	31.2 (27.0–50.6)	29.9 (27.0–54.4)	34.3 (26.1–49.8)
**Waist circumference (in)**	31.9 (28.8–38.2)	38.1 (33.9–39.6)	30.1 (28.1–33.1)
**Total MVPA per week (min)**	218.5 (106.5–333.8)	235.0 (119.5–426.3)	207.0 (101.8–293.3)
**Total sedentary time per week (hours)**	76.3 (68.0–82.9)	67.0 (62.2–77.9)	79.5 (70.9–83.5)
**Average sedentary h/day**	11.1 (9.7–11.8)	9.6 (8.9–11.1)	11.4 (10.2–12.0)
**Total number of sedentary bouts per week**	133.5 (120.3–148.8)	125.5 (107.0–152.0)	135.5 (120.8–148.3)
**Average number of sedentary bouts/day**	19.1 (17.2–21.3)	17.9 (15.3–21.7)	19.4 (17.3–21.2)
**Average duration of sedentary bouts (min)**	22.7 (20.1–25.2)	20.2 (18.9–23.5)	23.3 (21.6–26.7)
**Total number of sedentary breaks per week**	126.5 (113.8–141.3)	118.5 (106.5–142.5)	128.5 (115.3–139.8)
**Average number of sedentary breaks/day**	18.1 (16.3–20.2)	16.9 (15.2–20.4)	18.4 (16.5–20.0)
**Average duration of sedentary breaks (min)**	27.8 (22.4–35.1)	35.5 (29.6–37.8)	25.1 (21.2–29.7)

All values are median (interquartile range) unless stated otherwise. Abbreviations: MVPA, moderate to vigorous physical activity.

**Table 2:** Sample Characteristics Stratified by Sedentary Time (hours/day).

Variable	Sedentary Time	
	<11.38 h/day *n* = 14	>11.38 h/day *n* = 18
	Frequency (% by Row)	
**Age (years)**		
25–34	7 (41%)	10 (59%)
35–44	0 (0%)	4 (100%)
45–54	4 (80%)	1 (20%)
55+	3 (50%)	3 (50%)
**Biological Sex**		
Male	7 (70%)	3 (30%)
Female	7 (32%)	15 (68%)
**Race/Ethnicity**		
White (including Hispanic White)	10 (40%)	15 (60%)
Black (including Hispanic Black) *	0 (0%)	0 (0%)
Other	4 (57%)	3 (43%)
**Education**		
Some college or less	4 (57%)	3 (43%)
College graduate	10 (40%)	15 (60%)
**Waist Circumference**		
Healthy	10 (38.5%)	16 (61.5%)
Unhealthy	4 (67%)	2 (33%)
**Physical Activity**		
Meeting/exceeding PA guidelines	11 (52%)	10 (48%)
Not meeting PA guidelines	3 (27%)	8 (73%)

Abbreviations: PA, physical activity. * No Black volunteers (including Hispanic Blacks) in this study.

**Table 3:** Prevalence Ratios for Sedentary Time as a Predictor of Prescription Medication Use Among Adults.

Variable	Model 1 PR (95% CI)	Model 2 PR (95% CI)	Model 3 PR (95% CI)
**Sedentary Time**	1.29 (1.08–1.55) *	1.70 (1.29–2.26) *	1.66 (1.25–2.19) *
**Age (years)**			
25–34		1.00	1.00
35–44		2.54 (1.10–5.87) *	2.61 (1.12–6.09) *
45–54		3.49 (1.57–7.73) *	3.86 (1.68–8.88) *
55+		0.34 (0.94–1.19)	0.35 (0.10–1.24)
**Biological Sex**			
Male		1.00	1.00
Female		1.25 (0.51–3.03)	1.22 (0.50–3.01)
**Race/Ethnicity**			
White (including Hispanic White)		1.00	1.00
Black (including Hispanic Black) ^‡^		-	-
Other		0.15 (0.04–0.51) *	0.16 (0.04–0.58) *
**Education**			
Some college or less		1.00	1.00
College graduate		0.45 (0.19–1.08)	0.44 (0.18–1.05)
**Waist Circumference**			
Healthy		1.00	1.00
Unhealthy		5.40 (2.40–12.15) *	4.28 (1.77–10.30) *
**Physical Activity**			
Meeting/exceeding PA guidelines			1.00
Not meeting PA guidelines			1.56 (0.77–3.14)

* *p* < 0.05. ^‡^ No Black volunteers (including Hispanic Blacks) in this study. Abbreviations: PR, Prevalence ratio; CI, Confidence interval; and PA, physical activity. Model 1, Crude; Model 2, Adjusted for age, biological sex, race/ethnicity, education, and waist circumference; Model 3, Adjusted for age, biological sex, race/ethnicity, education, waist circumference, and physical activity.

**Table 4:** Prevalence Ratios for Sedentary Bout Frequency and Duration as a Predictor of Prescription Medication Use Among Adults.

	Sedentary Bouts	
	Frequency	Duration
Variable	Fully Adjusted Model PR (95% CI)	Fully Adjusted Model PR (95% CI)
**Sedentary Time**	1.36 (1.12–1.64) *	1.09 (1.03–1.16) *
**Age (years)**		
25–34	1.00	1.00
35–44	2.62 (1.18–5.78) *	3.26 (1.44–7.38) *
45–54	4.76 (1.95–11.63) *	2.93 (1.26–6.81) *
55+	0.59 (0.16–2.21)	0.41 (0.11–1.50)
**Biological Sex**		
Male	1.00	1.00
Female	2.26 (0.89–5.73)	1.31 (0.51–3.39)
**Race/Ethnicity**		
White (including Hispanic White)	1.00	1.00
Black (including Hispanic Black) ^‡^	-	-
Other	0.22 (0.06–0.76) *	0.30 (0.09–1.01) **
**Education**		
Some college or less	1.00	1.00
College graduate	0.64 (0.27–1.48)	0.41 (0.17–1.01) **
**Waist Circumference**		
Healthy	1.00	1.00
Unhealthy	8.50 (2.62–27.63) *	1.65 (0.82–3.33)
**Physical Activity**		
Meeting/exceeding PA guidelines	1.00	1.00
Not meeting PA guidelines	2.41 (1.23–4.73) *	1.81 (0.89–3.67)

* Significance at *p* < 0.05. ** Borderline significance at *p* = 0.05. ^‡^ No Black volunteers (including Hispanic Blacks) in this study. Abbreviations: PR, Prevalence ratio; CI, Confidence interval; and PA, physical activity. Fully adjusted Model: adjusted for age, biological sex, race/ethnicity, education, waist circumference, and physical activity.

## Data Availability

The data presented in this study are available at the request of the corresponding author in order to keep the privacy of the participants.
